# Translational Initiative to Minimize Childhood Hemiparesis After Asymmetrical Perinatal Brain Injury

**DOI:** 10.3390/bioengineering13090981

**Published:** 2026-08-26

**Authors:** Susan V. Duff, Emily Garavatti, Terrie Inder

**Affiliations:** 1 Department of Physical Therapy, Crean College of Health and Behavioral Sciences, Chapman University, Irvine, CA 92618, USA; 2 Neonatology Division, Children’s Hospital of Orange County, Orange, CA 92868, USA; emily.garavatti@choc.org (E.G.); terrie.inder@choc.org (T.I.)

**Keywords:** asymmetrical perinatal brain injury, perinatal stroke, cerebral palsy, hemiparesis, intervention

## Abstract

The purpose of this perspective paper is to present a translational approach aimed at identifying the critical periods in brain development during which to provide neuroprotective and neurorestorative strategies, followed by prehensile training, to minimize hemiparesis and foster skill acquisition after asymmetrical perinatal brain injury (APBI). We contend that hemiparesis could be minimized after APBI through a multimodal sequential approach based on timely implementation of evidence-based intervention strategies. If APBI is identified early, neuroprotective techniques can be used in the first 10 days of life to minimize cell death. This can be followed in the next 3 months by the provision of timely neurorestorative strategies to enhance neuron survival and promote brain growth. Ideally these strategies will prime the system for targeted prehensile training from 3 months and beyond. Building on animal work, scientifically sound pilot work in human infants is needed to determine the best timing and therapy dose verified with sensitive outcome measures available before widespread implementation. Strategic planning among a multidisciplinary group working in basic science, medicine, and rehabilitation will inform efforts to bring this translational approach to clinical fruition.

## 1. Introduction

Asymmetrical perinatal brain injury (APBI) can be described as unequal or one-sided brain injury before, during, or within 4 weeks of birth. This condition is typically diagnosed in the NICU based on magnetic resonance imaging (MRI) and neurobehavioral signs. APBI can stem from perinatal arterial ischemic stroke (PAIS) in infants born at term and periventricular hemorrhagic infarction (PVHI) in infants born preterm [1,2]. White matter injury or parenchymal hemorrhages can also lead to APBI. Neonates who are at high risk for unilateral cerebral palsy (UCP) or one-sided weakness and incoordination, after APBI may present with injury to the corticospinal tract or basal ganglia/thalamus [3,4], leading to hemiparesis.

Hemiparesis secondary to APBI often impedes the development and execution of prehensile skills, including performance of upper limb reach to grasp tasks [3,4,5,6]. Historically, hand and arm function have been found to improve after intensive training in children with UCP and subsequent hemiparesis [7,8,9,10,11,12,13,14,15]. Yet, despite training, impaired dexterity and function often persist [12,16,17], suggesting that training may have been provided too late. To examine the benefit of earlier training, constraint-induced movement therapy (CIMT), bimanual training, and combined approaches have been examined in infants as young as 3 months corrected age [18,19,20,21,22,23], yet later outcomes for these children have not yet been determined. While a positive trend toward ongoing improvement is anticipated, it is also possible that even with early training, the neural substrate may be insufficient to allow full recovery [22,23].

We present a perspective paper that proposes that, to prevent or reduce childhood hemiparesis, there should be complimentary approaches with greater utilization of innovative strategies to prevent cell death and promote brain growth in early infancy before neuronal loss or impaired connectivity within the corticospinal system occurs. These strategies, applied in early infancy to promote neuronal survival and connectivity, can be followed by hand and arm training in infancy and childhood. The paper presents a translational multimodal approach aimed at reviewing the best time periods in brain development to provide neuroprotection and promote brain growth, followed by prehensile training, in an effort to minimize hemiparesis and foster skill acquisition after APBI in infants at risk for UCP.

## 2. Brain Development and Neural Injury

A multimodal approach aimed at minimizing hemiparesis after early brain injury requires close examination of the neural processes associated with postnatal brain development to determine which processes can be modified with specific intervention(s) and at what time point [23,24,25,26,27,28]. Select interventions focusing on the prevention of cell death or promoting brain growth have been supported in animal work, with some gaining support for use with human infants [29,30,31,32,33,34,35,36,37]. Moving key research from animal work to human infants is feasible through collaboration between professionals in basic research, medicine, and rehabilitation [38,39]. Innovative methods to promote brain growth are expanding [40,41,42,43].

### 2.1. Neural Processes Associated with Brain Development

Cerebral development occurs in an orderly systematic fashion [26,27,28,29,42,43,44], with the embryonic brain present by 2–3 months of gestation [26]. Neuronal proliferation and migration follow over the next 2 months, followed by neuronal organization and myelination that extend years postnatally. Some brain maturation and developmental processes continue into adolescence and adulthood [26,43,44,45]. Neural injury alters this trajectory and activates expansion of select cell populations [46,47,48,49,50]. Table 1 illustrates these key processes, including (1) proliferation, (2) migration, (3) synaptogenesis, (4) selective pruning, and (5) myelination [26,27,43,44,45]. In addition, critical events shown to be direct or indirect mediators of hemiparesis are highlighted. While not all undesirable consequences can be negated, some interventions may prevent apoptosis or neural degradation caused by these events, whereas other methods may foster neural growth and connectively.

Proliferation is the process in which neurons and glial cells are formed by symmetrical and asymmetrical division of neural progenitor cells [26,27]. This is a robustly active process that results in the development of nearly 100 billion nerve cells. Proliferation can be disrupted by genetic causes or teratogenic etiologies such as radiation, infections (e.g., rubella, cytomegalovirus, zika virus), or metabolic or toxic factors (e.g., alcohol, hyperphenylalaninemia) [27]. Disruption to proliferation most commonly results in microcephaly. Hemiparesis resulting from viruses has been reported in older children [50,51], though this stems from injury rather than disruption of proliferation.

Neural migration occurs when newly formed neurons are relocated from the ventricular and subventricular zones to the region where they will permanently reside and function [27]. The peak period for migration is 3–5 months of gestation, with the cerebral cortex and cerebellum being key structures formed during this process. When neurons fail to migrate to their proper location, malformations such as polymicrogyria, heterotopias, and cortical dysplasia result, which are commonly associated with the development of seizures [23]. Hemiparesis may result from malformations along the motor strip [52,53,54]. Krsek et al. [52] found that 25% to 37% of infants with focal dysplasia did develop hemiparesis. Seizures associated with focal dysplasia are typically treated with pharmaceuticals and, in refractory cases, surgery.

Synaptogenesis is the formation of functional connections (synapses) between neurons, is ongoing throughout life, and supports learning and neuroplasticity. A related process is neurite outgrowth in which the number of dendritic spines, axons, and synaptic contacts rises, contributing to central nervous system functional capacity [26,51,55]. Dendritic and axon length increase 5–6 fold in the first 6 months postnatally [26]. Apoptosis limits the number of neurons capable of synaptogenesis and neurite outgrowth, thus indirectly leading to hemiparesis. If neuroprotective strategies can minimize apoptosis in the neonatal period, this may enhance the CNS’s capacity for synaptogenesis.

Selective pruning is the phase in which unused, weak or obsolete connections are eliminated, while those that undergo repeated use are strengthened [56,57] which may be a key process in the development of hemiparesis. The ipsilateral corticospinal tract (CST) connections present at birth begin to be eliminated 6 months postnatally [58] so that by 24 months of age, many ipsilateral CST connections are gone. After unilateral perinatal injury to the primary motor cortex (M1), the risk of hemiplegia in the contralateral limb increases [59,60]. Outward signs of hemiplegia may be revealed once ipsilateral pathways from the intact M1 region begin to be eliminated, reducing the neural substrate. This period of elimination coincides with the expansion of age-appropriate prehensile skills. Training in unilateral and bilateral hand/arm use in early infancy, prior to and during selective pruning is critical to delay or minimize pruning of ipsilateral CST tracts.

Myelination is the process by which myelin, a fatty insulating sheath, begins to surround the axon [26,61], accelerating nerve conduction velocity. The neonatal brain is predominantly unmyelinated [62], with postnatal myelination occurring rapidly in the first year and continuing slowly into the 3rd decade [63]. Myelin is produced by oligodendrocytes, which are generated from preoligodendrocyte progenitor cells [64]. Preoligodendrocytes are sensitive to oxidative and inflammatory injury, leading to impaired myelination [40,49,65]. Preoligodendrocyte injury leading to dysmaturation is a component of underlying neuropathologic process seen in preterm white matter injury, including periventricular leukomalacia (PVL), which can lead to hemiparesis [66,67,68]. Tracking of brain development has been done with neuroimaging, adding valuable insight into select conditions [69]. Thomas et al. [68] used diffusion tensor imaging (DTI) to examine five children with unilateral periventricular white matter injury and unilateral cerebral palsy (CP). They found a significant reduction in CST fiber count linked with the paretic limb and evidence of CST reorganization associated with the non-paretic limb. Secondary degeneration was also found in the brainstem, corpus collosum, and deep brain nuclei linked with the paretic limb. Environmental enrichment in the neonatal period [70], including methods to foster motor skill learning, have been proposed as a way to increase the production of oligodendrocytes. The methods reviewed below, such as positive sensory exposure [41,70,71] and training using the paradigm of contingent reinforcement [72,73,74], may help foster the production of oligodendrocytes and subsequent myelination through activity-based regeneration.

### 2.2. Neurotransmitters and Networks

Brain development relies on the influence of neurotransmitters and the strengthening of neural networks. Abundant neurotransmitters available at birth aid the neonate’s transition to extrauterine life and are crucial to activity-dependent plasticity [75]. Glutamate regulates neurogenesis, neurite outgrowth, synaptogenesis, and apoptosis in the developing brain [76]. Brain-derived neurotrophic factor (BDNF), glial cell line-derived neurotrophic factor and other growth factors are influenced by glutamate. BDNF levels increase from infancy to young adulthood and correspond with structural maturity [77]. Full-term neonates [78] and breastfed infants [79] have higher BNDF levels. The period of significant synaptogenesis, 2–4 months postnatally, may be the best time to influence BDNF production and synaptic plasticity through environmental enrichment [70,80] in preparation for prehensile training. As with adults, BDNF levels may increase in response to motor activity [81], but this needs further study in young infants.

### 2.3. Remodeling

Remapping of the motor or somatosensory cortex and re-establishment of vital neural networks are key factors in recovery in infants with APBI [33,62,82]. Yap and colleagues [62] examined brain development from 2 weeks to 2 years of age with diffusion tensor imaging (DTI) using a three-dimensional modeling technique, fiber tractography. They found that small local networks evolved into large, distributed networks that operate in a functional manner. Insufficient control of spinal motor circuits [31,60] may account for why signs of hemiparesis become apparent late and evolve over the first 2 years of life [60,83]. Evidence from Friel et al. [33] suggests that tailored prehensile training programs in a feline model conducted in a timely manner promote cortical mapping and repair of spinal motor circuitry to re-establish neural networks. To enhance success, motor training should be introduced in early infancy to induce activity-based competition for neural resources between the CSTs of both hemispheres to prevent childhood hemiparesis [33,84,85].

## 3. Translational Multimodal Approach to Minimize Hemiparesis

**Figure 1:**
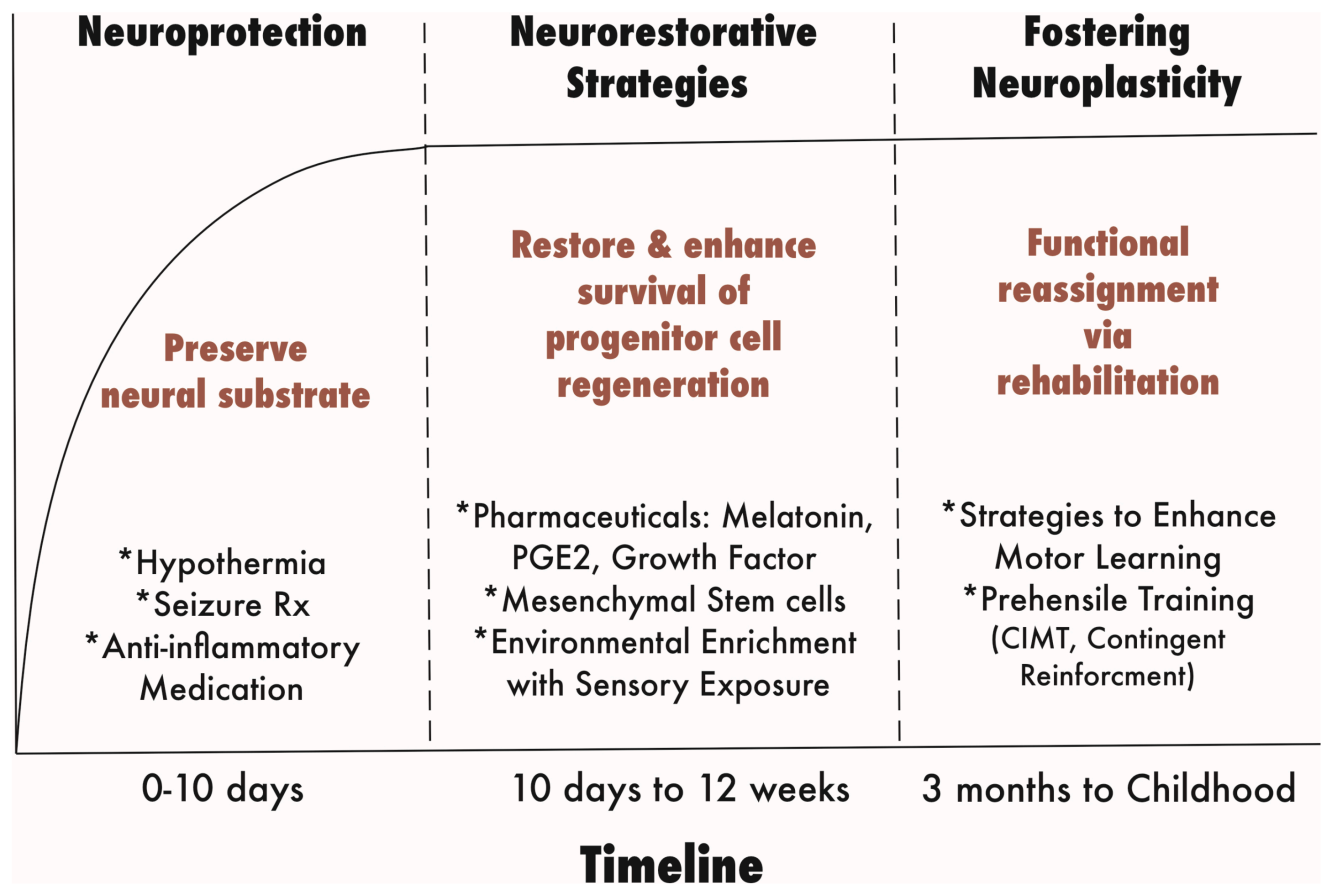
Multimodal approach timeline from birth to childhood.

A translational multimodal approach based on evidence would include three key areas: (1) neuroprotection; (2) neurorestoration; and (3) prehensile training (Figure 1). A combined approach optimizing neuroprotection, neurorestoration with brain growth, and motor training is vital, given that up to 50–70% of infants who sustain perinatal arterial ischemic stroke (PAIS) later display evidence of upper limb hemiparesis [86,87,88]. Since significant synaptogenesis occurs from about 28 weeks’ gestation to 3 months postnatally, it is crucial to preserve as many cells and synapses as possible to ensure that the infant retains sufficient neural substrate needed for brain growth and prehension development. A multimodal approach would require collaboration among professionals from birth through the first year of life and beyond.

### 3.1. Neuroprotection

Preserving the integrity of the cerebral mantle of newborns after injury is essential. Different approaches are necessary to address the varied etiologies of brain injury that occur in the preterm vs. the term infant. Here we review non-pharmaceutical and pharmaceutical modes of neuroprotection which vary regionally and by age of the infant. Suggested non-pharmaceutical methods used in most NICUs include therapeutic hypothermia, adequate nutrition, physiologic support, and kangaroo care. Recommended pharmaceuticals progressively change as research findings are published. All methods are now reviewed.

Therapeutic hypothermia. This form of neuroprotection is considered standard of care in term-born newborn infants with hypoxic-ischemic encephalopathy (HIE) to prevent apoptosis or cell death [89,90,91]. Immediately after an episode of hypoxia-ischemia, the neonatal brain enters a phase of “primary cell injury” [91] in which high-energy metabolites are depleted, cells begin to swell and extracellular amino acids accumulate. In the next “latent phase” there is recovery of cerebral circulation and oxidative metabolism. Yet 6 to 15 h after the initial episode of hypoxia-ischemia, secondary deterioration may begin as noted by seizures, resumed cell swelling, inflammation, abnormal receptor activity and apoptosis. Hypothermia administered within 6 h of birth or during the “latent phase” of HIE can lead to a reduction in the incidence of neurological deficits and long-term disability stemming from secondary deterioration [91,92].

**Figure 2:**
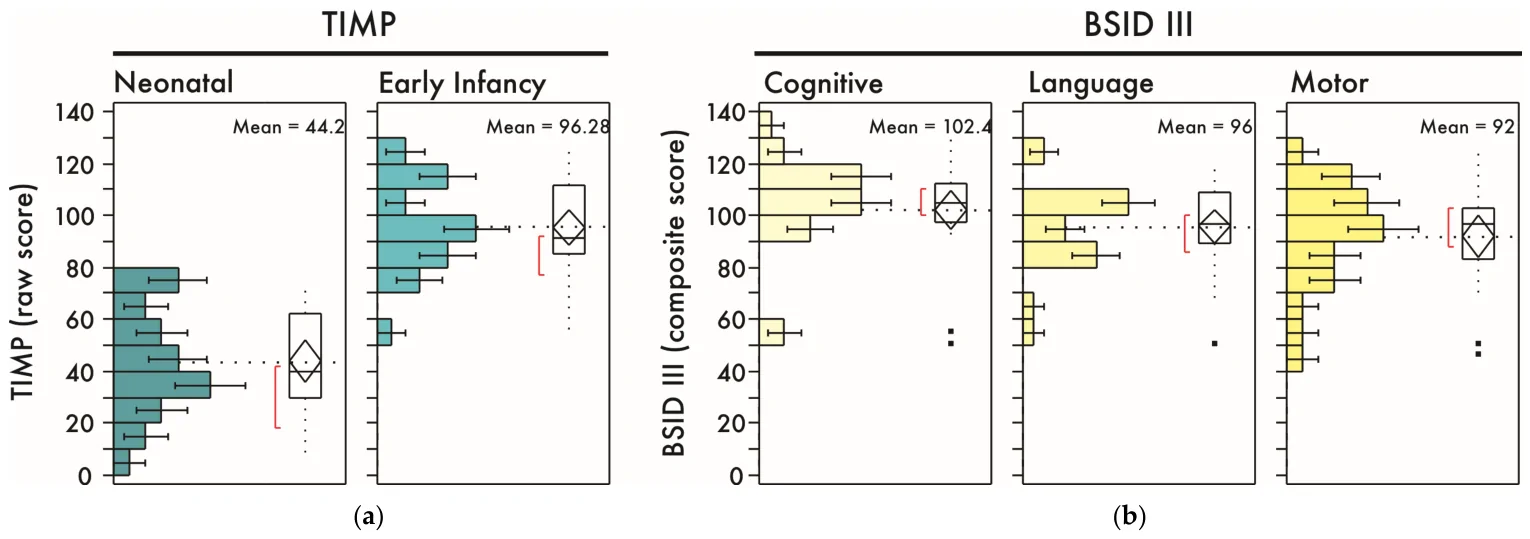
Study with HIE and hypothermia: (**a**) CoolCap^®^ in place on the head of a full-term infants with HIE. The cap provides a form of hypothermia for 72° in infants with HIE when placed within 6° of birth. (**b**) Developmental outcome with TIMP results on the left from the Neonatal and Early Infancy time periods (blue) and results from the BSID III on the right (yellow). The improvement in sensorimotor outcome from the neonatal period to early infancy based on the TIMP was maintained at one year of age based on the BSID III [93].

We assessed developmental outcome after hypothermia treatment with a CoolCap^®^ in infants with HIE [93] during the neonatal period and early infancy (2–4 months of age) based on the Test of Infant Motor Performance (TIMP) [94] and again at one year of age with the BSID III [95]. As shown in Figure 2, we found neuromotor performance improved based on the TIMP with 32% scoring in the average range in the neonatal period and 88% scoring in the average range by 2–4 months. By one year of age, 92% of infants performed in the average range on cognitive and language subtests and 76% were in the average range on motor subtests of the BSITD III. Retention in neuromotor performance from early infancy to one year of age implies that hypothermia may have prevented apoptosis, preserving neurons and synaptic connections and allowing sufficient development of neural networks.

The onset of injury in HIE during the birth process allows timely use of therapeutic hypothermia within the first 6 h of birth for maximal efficacy [90]. Yet most newborn infants with an APBI or stroke are not diagnosed immediately after birth, therefore missing the window for hypothermia to be effective. One study examined the effect of whole-body hypothermia in a small group of infants with perinatal stroke [96]. The authors found that five out of 15 infants diagnosed with perinatal stroke based on MRI received hypothermia and did not show evidence of seizures in the neonatal period whereas seven of 10 untreated infants did. While limited studies have investigated hypothermia following APBI [39,96,97], it is not considered standard practice and thus requires further investigation.

Adequate nutrition. Digestion of key nutrients is an essential component of fetal growth and neonatal care. It provides the foundation for ongoing brain development, health, and neuroplastic changes [98]. Optimal nutrition can support ongoing development of neural processes from proliferation to myelination [99]. Essential nutrients with a neuroprotective influence for the neonate with brain injury include docosahexaenoic acid (DHA), choline, iron, and protein [100,101,102,103]. These nutrients support gray and white matter growth, prevent injury to cells and promote development in the neonatal period [98,99,102,103,104,105,106,107]. Neonates can receive maternal milk, donor milk, or formula orally or through gastric systems [108]. Some infants may require parenteral nutrition which bypasses the digestive system. Fortunately, infant formula is designed to supply sufficient nutrients to neonates and infants who sustain APBI [109,110,111]. This area of clinical research is progressing rapidly; thus, nutrition should continue to play a strong role in neuroprotection of infants at risk.

Physiologic support. Maintaining good neuro-supportive care includes normotension, normoglycemia, normocarbia [49,112], and avoidance of physiologic swings, as undertreating and overtreating physiologic disturbances are associated with worse outcomes in those with brain injury and in the preterm population.

Kangaroo care. To achieve skin to skin contact or kangaroo care, mom or dad holds their baby upright, belly-down directly against their bare chest, while the infant wears only a hat and diaper, covered in a blanket for warmth. It was developed from the work of Edgar Ray, MD and Hector Martinez, MD from Bogotá, Colombia [113,114]. Kangaroo care (KC) has been found to stabilize an infant’s vitals, reduce stress, and release oxytocin for bonding [115,116]. An extension of KC is kangaroo mother care (KMC) which also includes exclusive breastfeeding, early discharge, and close follow-up [115]. The benefits of KMC beyond KC include a reduction in neonatal sepsis, hypothermia, hypoglycemia, lower mean respiratory rate and pain, higher oxygen saturation, temperature, and head circumference growth [117,118]. Implementation of KC or KMC varies depending on the setting, sociocultural context, and constructs of parental roles. In addition, other responsibilities and stressors can influence the ability of the staff and parents to carry out KC or KMC successfully in the NICU and home [119].

Pharmaceutical therapies have been investigated for use in neonatal stroke, white matter injury of prematurity, intraventricular hemorrhage, and as an adjunct to therapeutic hypothermia in HIE for their neuroprotective effects, though none are clinically available at this time. Common targeted mechanisms include anti-inflammatory (e.g., azithromycin), anti-apoptotic, and anti-excitatory, with some agents having multiple mechanisms of action (e.g., melatonin, erythropoietin). See Table 2 for a list of pharmaceuticals. Injury-specific mechanisms and targets are also being explored, such as fibrinolytics to dissolve clots and restore blood flow in acute ischemic stroke, as is currently done in adults, and to dissolve clots in post-hemorrhagic ventricular dilatation. Iron chelation is being explored in intraventricular hemorrhage. The goal is to minimize tissue loss after injury. The preclinical evidence of these pharmaceuticals provides hope, but their benefit has yet to be established in human clinical trials. The list of potential pharmaceuticals continues to expand, yet further research is needed to prove their safety and efficacy in human infants and to determine the optimal timing and dosing frequency and duration [120,121,122,123,124,125].

### 3.2. Neurorestorative Strategies

After perinatal neural injury, methods to restore the neural substrate are of interest. Animal studies reveal that select pharmaceuticals and other interventions contribute to brain growth and repair. While some procedures are supported with research findings, others require further investigation. Select methods will be reviewed here.

Pharmaceuticals to promote neuronal repair are being explored in both adult and infant brain injury; none are clinically available yet [121,126]. These agents act by promoting neurogenesis, angiogenesis, and synaptic plasticity. Many of them also have neuroprotective properties (e.g., erythropoietin). Biologics are also being explored, including stem cells, exosomes, and growth factors. There are additional agents like hydrogels that are in preclinical investigation and can serve as substrate for delivery of therapies in addition to providing physical support, preventing cyst formation, and encouraging regrowth. In addition, preclinical work is exploring the targeting of innate pathway activation to promote oligodendrocyte precursor cells after injury to improve myelination [35,70]. Further study is needed to determine the optimal intervention, route of administration, timing, dosing, and frequency to promote neurorestoration after perinatal brain injury. See Table 2 for summary of pharmaceuticals and other agents being explored for neurorestoration.

Environmental enrichment. In preclinical work, enrichment of the environment has been shown to contribute to neural recovery. While brain injury delays the sequential development of oligodendrocytes, important to the production of the myelin sheath, Gallo and colleagues [70] have shown that environmental enrichment (EE) promotes myelination through endogenous processes. In the rat model, exposure in a natural habitat with novel stimuli, social interactions, and voluntary physical activity are encouraged [116].

Clinicians working with newborn infants have drawn upon the preclinical work of Gallo and colleagues [127,128,129] and propose that there are critical periods and elements essential to enriching the environment. They suggest that EE should begin immediately after birth with positive sensory exposure [71,128] as reviewed below and continue through later infancy and childhood. Importantly, all components are necessary to promote functional recovery (motor, sensory, and cognitive) [129]. Current early interventions may not be optimal since they do not specifically target the risk each particular infant has. Imaging could be used to individualize the intervention, so that it is adequately directed and target specific risk factors prior to onset of symptoms. As suggested in this paper, targeting motor therapies for those at risk for hemiparesis (injury to PLIC, basal ganglia or motor cerebral cortex) or vision therapy for those at risk for cortical vision impairment (injury to occipital lobes or optic radiations) is critical. The authors recommend that rehabilitation promoting motor learning, active movement, engagement of parents, and modifications to the environment may be best to foster early achievement of skill.

Positive sensory exposure. An association has been established between positive sensory exposure and brain growth [36,71,130]. The ideal NICU environment has yet to be fully recognized, as private rooms limit sensory stimuli for infants, including both noxious noise and developmentally supportive exposure to human voice, such as language exposure [131,132], highlighting that the ideal NICU environment may vary based on social circumstances, including family availability [133]. Exposure to noxious stimuli like painful procedures remains high, thus the need for positive sensory stimulation is vital. Pineda et al. [41,71] examined and implemented a program entitled SENSE—supporting and enhancing neonatal intensive care experiences. This program provides evidence-based multi-sensory experiences for infants at risk while engaging parents in the process. Interventions focus on rocking for vestibular input, soft music for auditory input, and holding with skin-to-skin care and massage as further reviewed below. Initial findings from this program conducted with preterm infants support improvements in parent confidence, neurobehavior, feeding, and communication at one year of age. The SENSE program has been implemented worldwide and studies providing further support are ongoing. Such interventions in the setting of APBI have not been fully established.

Massage is a positive exposure that may influence brain growth and neural connectivity [130,134]. Guzzetta et al. [130] conducted massage for 10 days (with a 2-day interval), 3 times/day, in 10-day-old preterm infants 30–33 weeks’ gestation. They found that massage modulated the level of endogenous or insulin-like factors (IGF-1) that aid in the regulation of brain growth and improved visual acuity by 3 months of age when compared to controls (45% greater). Although the authors found that by 7 months of age, visual acuity was similar between the groups, the rate of change and the period of heightened arousal may have had positive benefits on brain growth and function.

The effects of positive sensory exposure are short-lived by their nature. However, the sensory experience may increase state regulation [36], helping to prepare the CNS and neuromuscular system for prehensile training. Evidence suggests that attentive infants in the awake-alert state learn more quickly than infants in any other level of state control [36,135]. Methods such as massage may serve to modulate levels of neurotransmitters and promote an awake-alert state as described in the Neonatal Behavioral Assessment Scale [136], in preparation for interventions after neural injury. While evidence-based sensory supports have not been specifically studied in infants with APBI, these methods warrant further investigation to see if these techniques alter sensorimotor behavior.

### 3.3. Neuroimaging Identification of Risk for Hemiplegia

Identification of the population at risk for hemiplegia is key for determining who should receive targeted therapy. As discussed above, hemiparesis is associated with asymmetrical brain injury, which may be vascular or infectious, but hemiparesis can also occur in the setting of developmental malformations, such as cortical dysplasias, along the motor tracts. MRI findings involving the posterior limb of the internal capsule and/or basal ganglia and/or cerebral cortex after perinatal arterial ischemic stroke are predictors for later hemiparesis [137,138].

### 3.4. Prehensile Training

Prehension involves locating, reaching, grasping, manipulating and releasing toys or objects of any kind. Prehensile training could focus on any of those components, yet for the purpose of this paper we will be focusing on reaching and grasping with the upper limb. We will introduce successful animal and human models of CIMT and then the paradigm of contingent reinforcement.

Targeted prehensile training programs aimed at strengthening neural connectivity and motor control reportedly prevent hemiparesis in the animal model and are slowly being translated to human infants. Animal models have also shown that we can take advantage of activity-dependent competition for neural resources to aid neural recovery post-injury [33]. Specifically, the integrity of the contralateral CST and the associated spinal circuitry from the impaired primary motor cortex (M1) could be reinforced through timely skill training to prevent the development of dominant connections from the intact M1, thereby enhancing contralesional motor function [139]. Further, evidence suggests that skill training can be used to positively reinforce redundant corticospinal pathways available before selective pruning subsides [56,60].

**Figure 3:**
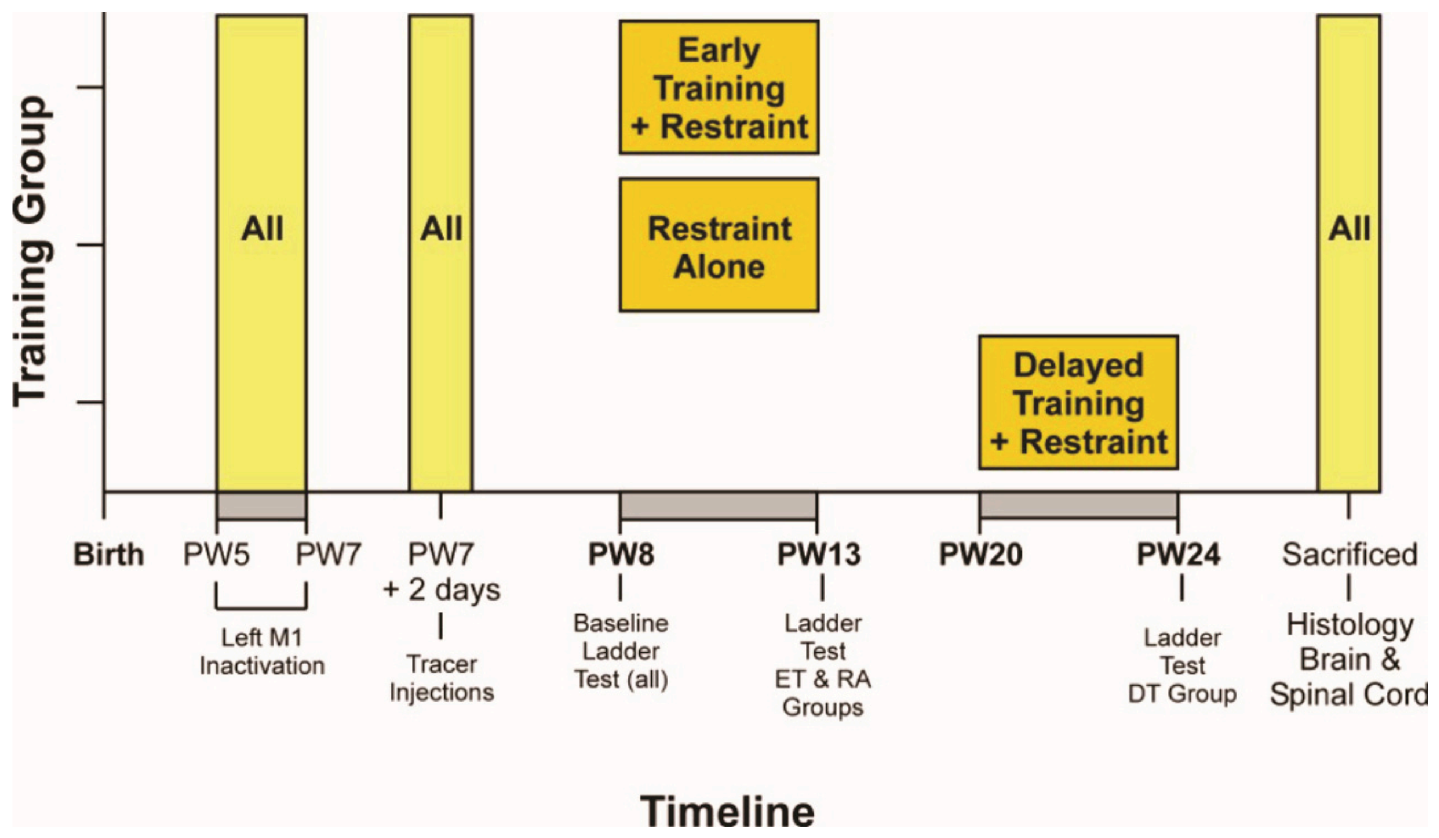
The timeline for the experimental design used in the training study with cats [33]. During weeks 5–7, the left M1 was inactivated and a tracer was injected 2 days later to track the CST connections. All three groups wore a restraint for 4 weeks. The early training plus restraint group and the restraint-alone group began at PW 8 and ended at PW 13. The delayed training group began at PW20 and ended at PW 24. All of the cats were sacrificed to examine the brain and the spinal cord histologically. PW = postnatal week; ET = early training; RA = restraint alone; DT = delayed training.

**Figure 4:**
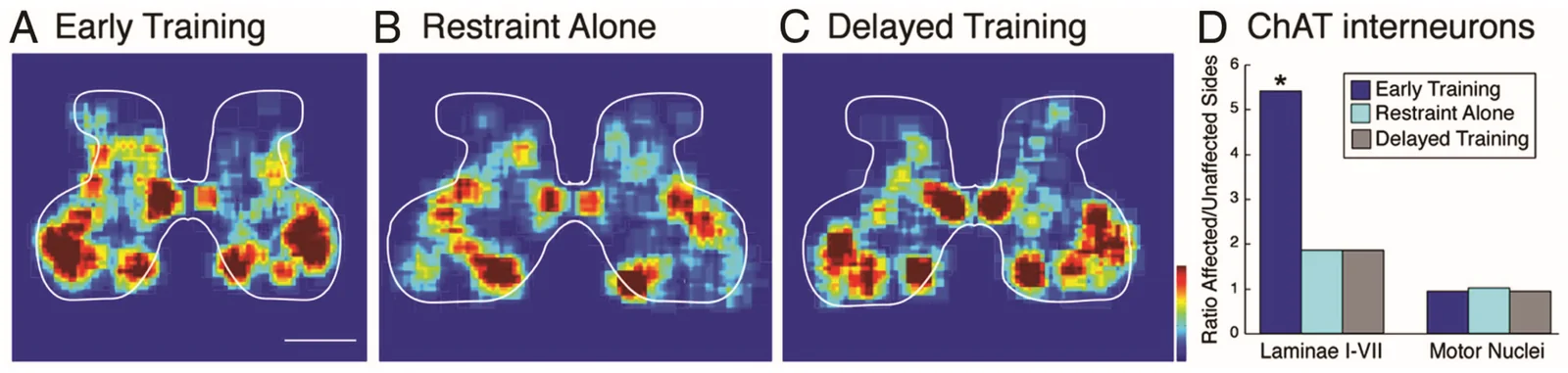
ChAT-positive cells distributed in the lower cervical spinal cord. (**A**–**C**) Density maps in the cervical enlargement for ChAT-positive cells. Motoneurons are predominantly depicted by the large dense clusters of cells in the ventral horn. For all panels, the color scale is the same (red_1.25_10 -5 cells/square_m). Scale bar, 1 mm. (**D**) Ratios of ChAT label on the affected side of the spinal cord (**left**) to the contralateral (**right**). The early training group had a much greater ratio of ChAT-positive cells in the intermediate zone (laminae I–VII) on the affected/trained side than the untrained side. The two sides of all groups were symmetrical with regard to the ratio of ChAT label in motor nuclei [33].

Constraint-induced movement therapy (CIMT). This form of prehensile training involves restricting movement of the upper limb that is not at risk for hemiparesis in a constraint that prevents movement, while the hand and arm at risk are encouraged to perform motor actions such as reaching and grasping. The feline model of prehensile training from Friel et al. [33] is an excellent template from which to design translational studies. Friel et al. [33] used CIMT to aid in the neural repair and restoration of function following inactivation of M1 between postnatal weeks (PW) 5–7. They compared the outcome of early vs. late 4-week-long training in a forelimb reaching task, during which kittens wore a restraint on the ipsilesional forelimb, with those of a control group that only wore a restraint and did not receive training (see Figure 3). The early training and control group wore the restraint from PW 8–12. The delayed training group wore the restraint from PW 20–24. As shown in Figure 4, the results indicate that early training restored CST connections and the M1 motor map, increased the number of cholinergic spinal neurons contralaterally, and prevented impairment in forelimb motor control during reaching and horizontal ladder walking. Delayed training restored only connections of the CST and the motor map of M1, and only CST connections were restored in the control group. Reportedly, the restraint alone was insufficient to recover the M1 motor representation [33]. As demonstrated in the primate stroke model [140], training was deemed essential to restore spinal circuitry, aided by an increase in choline acetyltransferase expression in spinal interneurons. Thus, restoration of CST connectivity and the M1 motor map and an increase in the numbers of cholinergic spinal interneurons are critical for gaining motor control and function following injury to the corticospinal system.

Studies examining the benefits of CIMT and bimanual training have found these training programs to be efficacious for children 6 to 20 years of age [11,12,13,14,15,16,17,20,21]. Research examining CIMT and bimanual training in infants less than one year of age [18,22] has shown that improvements can be made if the dosage is high enough. Evidence reveals that the potential to develop useful prehensile skill may be greater if training is conducted at younger ages [18,19,20,21,22,141]. This potential may be even greater if training is initiated by 3 months of age given the dynamic and redundant neural processes present in early infancy before selective pruning begins.

Contingent reinforcement. Self-initiated reaching is one of the hallmarks of early infancy. Typically developing (TD) infants initially display flapping movements, yet by 3 to 5 months of age these movements are refined to purposeful reaching [142]. One reward infants can receive for reaching successfully is having the arms activate a musical mobile or toy [72,73,74]. By 3 to 4 months of age, TD infants display signs of associative learning and memory in the paradigm known as *contingent reinforcement (CR)*, in which an infant’s leg is tethered to an overhead mobile such that leg kicks result in proportional mobile movement [73,74]. In the study by Heathcock et al. [73], the authors studied the benefit of CR on TD and preterm infants and found that preterm infants did not show the associative learning, as noted by an increase in kicking rate, found in TD infants. They suggested that a suboptimal arousal level may have influenced the findings and that future work may determine the best timing and form of CR for this group of infants.

With advances in technology, methods to employ the CR paradigm can now be tailored to the primary dysfunction an infant at risk presents with and provide higher dosage and intensity of targeted intervention. Using surface electromyography, we have shown that muscle activation above a pre-set threshold could trigger a video to play in infants who sustain a birth-related injury to the brachial plexus [72]. While this system is effective for infants with limited muscle activation, some infants tend to disuse the arm(s) or display maladaptive patterns. For them, a movement-based system may be easier to learn and could be more effective. Sargent and colleagues [143,144] used a three-dimensional motion analysis system to quantify the spatial exploration of the feet, as they attempted to activate an overhead mobile. Infants in those studies were typically developing (TD) at 4 months of age [143] and preterm at 4 months corrected age [143]. Both groups increased their spatial exploration, yet the preterm infants displayed less task specificity perhaps due to variations in perception, strength, and postural control. We have designed and tested a system of CR which uses wrist-based inertial sensors to trigger a mobile or toy to play when acceleration exceeds a set threshold [145]. This system has been found feasible for infants less than 6 months of age, including an infant who sustained a perinatal stroke.

Infants with APBI may need activity-dependent competition to enhance neural circuitry. Thus, combining CIMT with strategies involving contingent reinforcement may be an ideal way to induce repetitive reaching in the limb most at risk for hemiplegia, fostering motor learning in this group [18,19,22,33,84]. Because contingent reinforcement is an age-appropriate paradigm for young infants, and CIMT leads to gains in active movement, this combination may take advantage of neuroplasticity to foster early motor skills in infants with APBI.

#### 3.4.1. Practical Recommendations

A multimodal approach to minimize hemiparesis requires inclusion of all three components: neuroprotection, neurorestoration, and rehabilitation. It is feasible for medical and rehabilitation personnel to employ all three components as the infant moves from one setting to the next. For *neuroprotection*, it is recommended that therapeutic hypothermia [89,90,91,92,96,97] be used by NICU personnel if the diagnosis of APBI is made early enough. In addition, it is essential to provide sufficient physiologic support [112] and administer targeted pharmaceuticals [120,121,122,123,124,125] during the neonatal period. We also recommend that adequate nutrition be provided immediately by NICU personnel and continued by parents in the home [98,99,100,101,102,103,104,105,106,107,108,109,110,111]. Kangaroo care is another method of neuroprotection that could be initiated by parents with guidance from NICU staff then continued in the home after discharge [113,114,115,116,117,118]. In terms of *neurorestoration strategies*, we highly recommend ongoing administration of key pharmaceuticals [120,121,122,123,124,125] and provision of environmental enrichment with positive sensory exposure as outlined in the SENSE program [127,128,129,130,131,132,133,134]. SENSE techniques begun by NICU personnel and parents include positioning to allow limb movement, simple massage, easy rocking, soft singing, or playing of classical music. Positioning the infant in right and left sidelying, as well as supine with receiving blankets for support, should allow movement of the upper and lower limbs most at risk for hemiparesis without limitations. As upper limb active movement increases, CIMT could be employed by lightly restricting motion in the arm not at risk while fostering reaching in the arm at risk. These reaching or reach-to-grasp actions could be done with the infant in sidelying so gravity is minimized as the infant attempts to reach toward a mirror secured to the crib rails or nearby toys. In the supine position, the infant would be moving against gravity, as the infant attempts to reach for hanging toys. The key to success is to ensure that the infant is attentive and capable of moving the arm and leg at risk since repetitive, purposeful action enhances neuroplasticity and motor learning. As this translational approach becomes more utilized in the NICU and the home, we may begin to see signs of hemiparesis lessening in infants with APBI.

#### 3.4.2. Case Example

**Figure 5:**
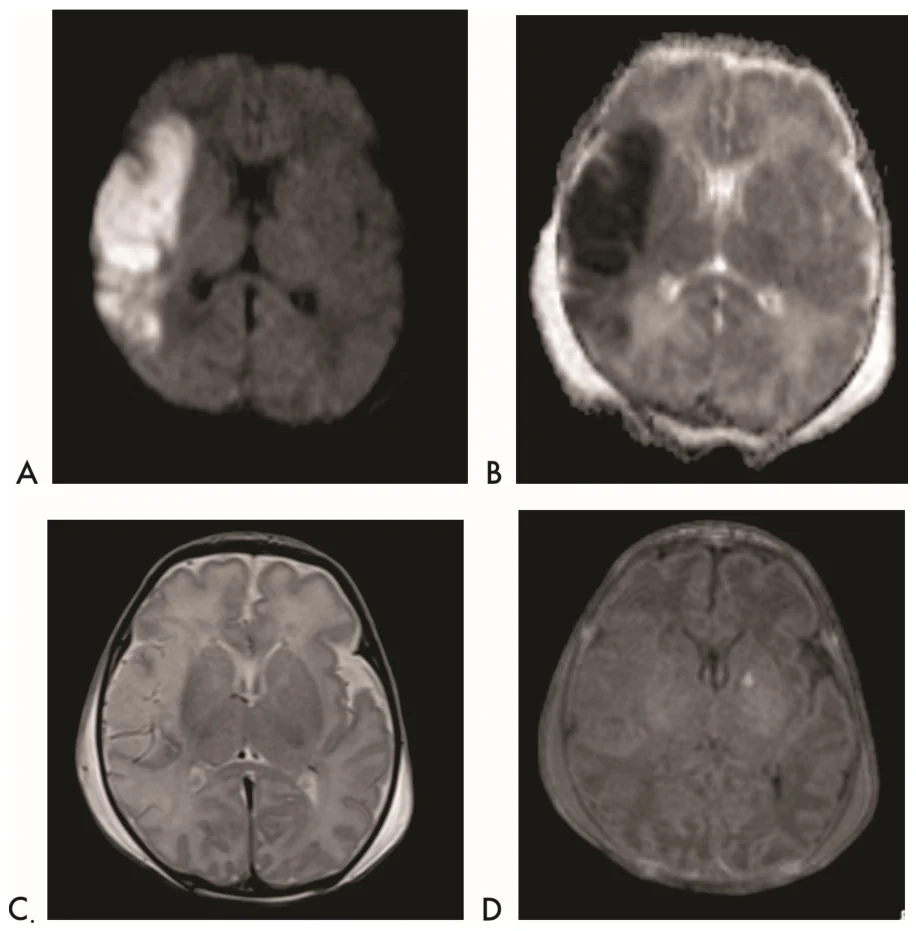
Case: MRI day 4. (**A**,**B**) diffusion-weighted images (**A**. SB100 and **B**. ADC) demonstrating right middle cerebral artery territory infarct. (**C**) T2 image demonstrating corresponding edema. (**D**) T1 image with hyperintensity in the left basal ganglia, most consistent with hemorrhage.

**Figure 6:**
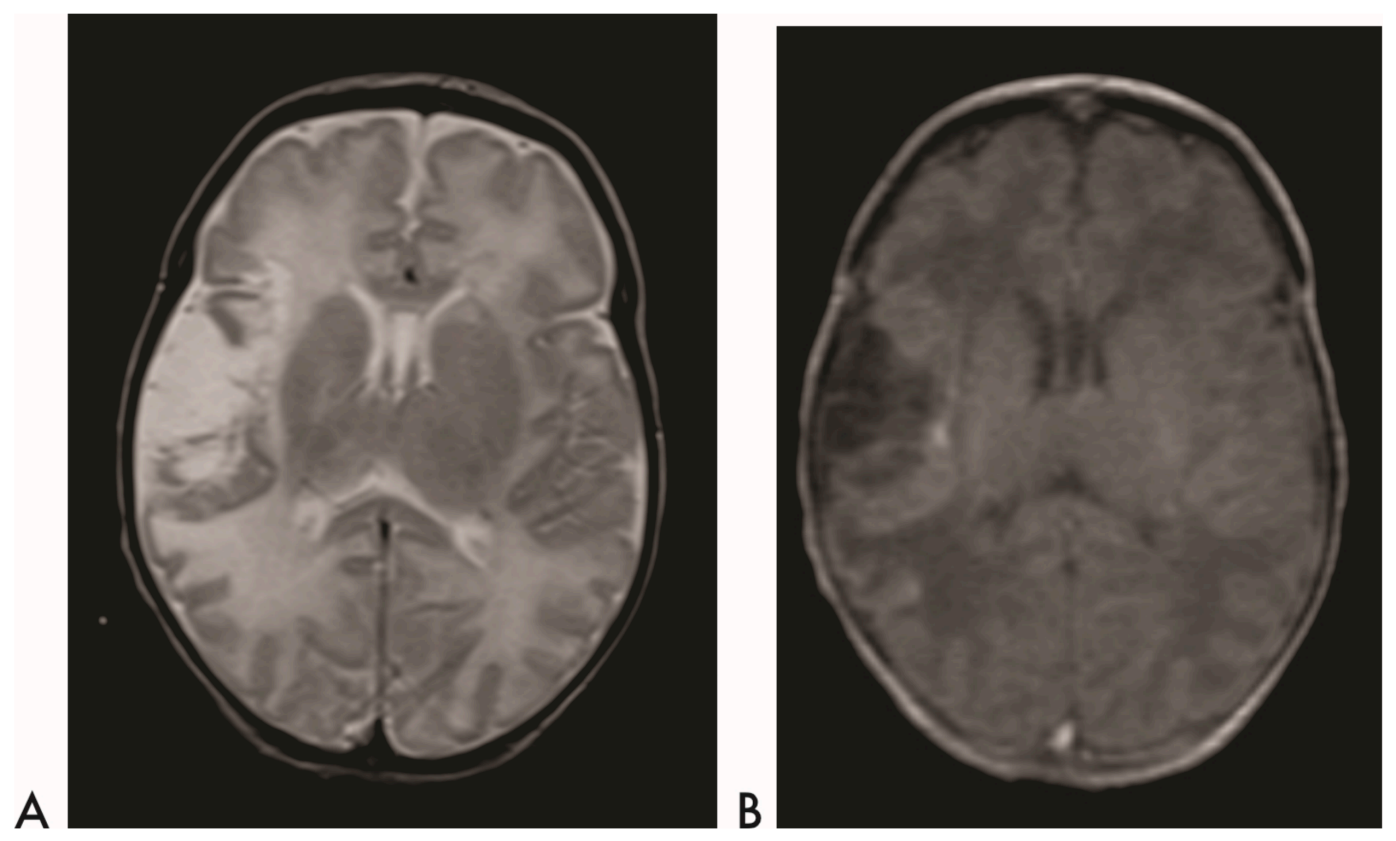
Case: MRI day 17. (**A**). T2 imaging demonstrating evolution of the right middle cerebral artery infarct with encephalomalacia. (**B**). T1 imaging demonstrating development of right-sided laminar necrosis vs. microhemorrhage and resolution of previously seen left basal ganglia hemorrhage.

Our group worked with an infant who sustained perinatal ischemic stroke in the right middle cerebral artery (MCA) territory and experienced all three phases of our proposed intervention to minimize hemiparesis: neuroprotection in the neonatal period, neurorestorative strategies in the NICU, and contingent reinforcement at about 6 months of age. Thus, he may serve as a great example of how this program could benefit infants at risk. This case infant was born at 36 weeks and 1 day by emergency c-section for a drop in fetal heart tones and failed vacuum delivery. The Apgar scores were 1, 3, and 4 at 1 min, 5 min, and 10 min of life. Neuroprotection provided in the form of therapeutic hypothermia was provided for 3 days. Seizures were noted arising from the right central area on days 1 and 2 of life and responded to phenobarbital. The infant was transitioned to levetiracetam monotherapy for maintenance anti-seizure medication. An MRI following rewarming on day 4 demonstrated diffusion restriction in the right MCA territory, while follow-up imaging on day 17 demonstrated evolution of the infarct with encephalomalacia (Figure 5 and Figure 6). Neurorestorative strategies in the form of positive sensory exposure were provided starting in the NICU and continued in the home by his parents and included reading to him, skin-to-skin contact, and visual stimulation. At one month of age, he was weaned off levetiracetam and began outpatient physical and occupational therapy, each twice a week. At about 6 months of age, as part of a training study using the paradigm of contingent reinforcement, he demonstrated the ability to repetitively activate an overhead mobile with his arm at risk for hemiplegia. Now at 2 years of age, he is developing well with equal use of both hands, and fine/gross motor skills within normal limits for his age (Bayley-4 motor standard score of 96, in the 39th percentile). He wears glasses for astigmatism and is receiving speech therapy for stuttering.

## 4. Conclusions

The methods to provide neuroprotection early after APBI followed by neurorestorative strategies may preserve and enhance the neural substrate essential to recovery. With this neural base, the CNS could be ready to refine the sensorimotor map and neural circuitry to minimize hemiparesis, allowing skill development [138]. The results from Friel et al. [33] suggest that to minimize hemiparesis and foster prehensile skills, training must begin early postnatally, well before the initiation of neuronal pruning in human infants at 6 months [27,58]. To enhance the long-term success of targeted training, our proposed translational multimodal approach may be best (see Figure 1). Yet are we ready for this exciting and important translational research opportunity? We propose that preliminary evidence is there, and it may be time to investigate the efficacy of this multimodal approach.

## Figures and Tables

**Table 1:** Association of neural processes with hemiparesis.

	Definition	Peak Time Period	Conditions Which Lead to Hemiparesis
**Neural** **Proliferation**	Neurons & glial cells formed by symmetrical & asymmetrical division of neural progenitor cells	Neuroblasts 5–25 weeks of gestation; Glial cells 20–40 weeks of gestation	Infections may contribute
**Neural** **Migration**	Relocation of newly formed neurons from ventricular & subventricular zones to brain area where they will permanently reside, given their resultant function	3–5 months of gestation	Malformations, such as cortical dysplasia, in the motor cortex
**Synaptogenesis**	Formation of new synapses. An associated process is neurite outgrowth in which the number of dendritic spines, axons & synaptic contacts increases	28 weeks of gestation to 3 months postnatally	Neural injury to the corticospinal system after synaptogenesis may limit recovery thus contribute to hemiparesis
**Selective** **Pruning**	Process in which weak & obsolete neurons are eliminated while those that undergo repeated use are strengthened	Begins 6 months postnatally for cortical spinal tracts	Unilateral injury to motor pathways; pruning of pathways from intact motor cortex reveals outward signs of hemiplegia with the onset of reaching
**Myelination**	Process in which myelin, a fatty insulating sheath, begins to surround the axon	Primarily in the 1st year postnatally; extends until the 3rd decade	Early insults to white matter impair pre-myelinating oligodendrocytes, resulting in periventricular leukomalacia

**Table 2:** Neuroprotection and neurorestorative pharmaceuticals and other interventions.

Therapy	Neuroprotective						Neuro- Restorative	Population	Phase of Study
	AE	AA	AI	AO	IC	Fib			
Tissue plasminogen activator [120,121,122,123]						✕		Neonatal stroke IVH	Unable to provide due to timing constraints Mixed results
Urokinase [120]						✕		IVH	Phase I
Deferoxamine [120]					✕			IVH	Preclinical
Anakinra [120,122]			✕					Preterm	Phase I/II
Azithromycin [121,122]			✕					HIE Preterm	Preclinical
Minocycline [120]			✕					IVH	Preclinical
Allopurinol [121,124]			✕	✕				HIE	Phase III
Sildenafil [121]			✕				✕	HIE	Phase I
Melatonin [121,122,123,124]	✕	✕	✕	✕				HIE Preterm	Needs a large RTC Phase I
Erythro- Poietin [121,122,124]	✕	✕	✕	✕			✕	HIE Preterm	Failed to demonstrate benefit in both groups
Stem cell [121,122,123,124]	✕	✕	✕				✕	HIE Stroke Preterm	Phase I Phase II Phase I/II
Growth factors [123,124]			✕	✕			✕	HIE Stroke	Preclinical

AE = anti-excitatory, AA = anti-apoptotic, AI = anti-inflammatory, AO = anti-oxidative, IC = iron chelation, Fib = fibrinolytic.

## Data Availability

The original contributions presented in this study are included in the article. Further inquiries can be directed to the corresponding author.

## Abbreviations

The following abbreviations are used in this manuscript:

APBI	Asymmetrical perinatal brain injury
CIMT	Constraint-induced movement therapy
CP	Cerebral palsy
CNS	Central nervous system
CST	Corticospinal tract
PVL	Periventricular leukomalacia
DTI	Diffusion tensor imaging
BDNF	Brain-derived neurotrophic factor
HIE	Hypoxic-ischemic encephalopathy
TIMP	Test of Infant Motor Performance
BSITD III	Bayley Test of Infant Toddler Development, 3rd edition
MSCs	Mesenchymal stem cells
EPO	Erythropoietin
MRI	Magnetic resonance imaging
EE	Environmental enrichment
PLIC	Posterior limb of the internal capsule
NICU	Neonatal Intensive Care Unit
SENSE	Supporting and enhancing neonatal intensive care unit sensory experiences
IGF-1	Insulin-like Growth Factor 1
PW	Post natal weeks
TD	Typically developing
CR	Contingent reinforcement
MCA	Middle cerebral artery
APGARS	Appearance, pulse, grimace, activity, respiration
